# Gallbladder Agenesis and a Large Common Bile Duct Stone: A Case Report and Literature Review

**DOI:** 10.7759/cureus.89777

**Published:** 2025-08-11

**Authors:** Ana K. Mena, Humberto Quiroz, Cynthia Sánchez, Alejandro H. Oliva, Daniela Ávila

**Affiliations:** 1 General Surgery, Hospital General Regional No. 1, Instituto Mexicano del Seguro Social (IMSS), Querétaro, MEX

**Keywords:** biliary colic, cbd, cholangitis, choledocholithiasis, common bile duct, gallbladder agenesis

## Abstract

Gallbladder agenesis is a rare congenital anomaly that is often asymptomatic but may present with clinical features mimicking biliary tract disease. The absence of the gallbladder is frequently undetected by abdominal ultrasound, leading to diagnostic uncertainty and potential surgical dilemmas. Accurate preoperative diagnosis is essential to avoid unnecessary surgical exploration and iatrogenic complications. We report a case of a 67-year-old male who presented with acute cholangitis secondary to choledocholithiasis. Cross-sectional imaging (CT and magnetic resonance cholangiopancreatography) revealed no identifiable gallbladder, while endoscopic retrograde cholangiopancreatography was inconclusive due to the presence of a large common bile duct stone. Laparoscopic exploration of the biliary tract was performed, resulting in the extraction of a 20 mm stone and intraoperative confirmation of gallbladder agenesis. This case emphasizes the importance of considering gallbladder agenesis in the differential diagnosis of biliary symptoms and highlights the need for comprehensive imaging to guide appropriate management and avoid unnecessary surgical risk.

## Introduction

Gallbladder agenesis is a rare but well-recognized congenital anomaly, first described by Lemery in 1701. Its incidence is estimated at 10-65 cases per 100,000 individuals, although the true prevalence may be higher due to the largely asymptomatic nature of the condition [1]. Despite its benign natural history, approximately 50% of affected individuals eventually develop biliary symptoms [2,3]. This anomaly shows a female predominance (3:1 ratio) and is most commonly diagnosed during the third decade of life [3].

Gallbladder agenesis results from a failure of the gallbladder and cystic duct to develop during the embryologic period, typically around the fourth week of gestation [4]. In some cases, it may be associated with other congenital anomalies or syndromes, such as trisomy 18, Klippel-Feil syndrome, and xanthomatosis [5]. Familial clustering has also been reported, supporting the utility of screening in asymptomatic relatives [6,7].

Symptomatic presentations may include right upper quadrant pain (up to 90% of cases), nausea and vomiting (66%), intolerance to fatty foods (37%), jaundice (35%), and dyspepsia (30%). These symptoms are thought to arise from biliary dyskinesia or choledocholithiasis and may resemble postcholecystectomy syndrome [2].

Preoperative diagnosis is essential to avoid unnecessary surgical exploration and reduce the risk of iatrogenic bile duct injury during attempts to locate a non-existent gallbladder, which may be misinterpreted on ultrasound as scleroatrophic or contracted. In cases of diagnostic uncertainty, magnetic resonance imaging (MRI) or endoscopic ultrasound (EUS) is recommended. However, many diagnoses are made incidentally during laparoscopic exploration [1].

## Case presentation

We present a case of a 67-year-old male with a history of musculoskeletal malformation of the right thoracic limb, type 2 diabetes mellitus under medical treatment, previous left inguinal hernia repair, transurethral resection of the prostate, and choledocholithiasis. Three months prior, he underwent endoscopic retrograde cholangiopancreatography (ERCP) with sphincterotomy and biliary stent placement; however, detailed findings from that procedure were unavailable. No other relevant personal or family history was reported.

The patient presented to the emergency department with a 72-hour history of right upper quadrant abdominal pain, fever, jaundice, generalized weakness, and adynamia. On physical examination, vital signs were stable, and the patient was alert and oriented. Notable findings included generalized jaundice, dry mucous membranes, a soft and non-tender abdomen, and a positive Murphy’s sign. The remainder of the abdominal examination was unremarkable.

Laboratory investigations revealed leukocytosis and cholestatic liver enzyme abnormalities, including elevated total bilirubin predominantly due to direct hyperbilirubinemia, and increased alkaline phosphatase levels (Table 1). Other laboratory parameters were within normal limits.

**Table 1:** Laboratory findings upon admission to the emergency department. Summary of blood test results showing leukocytosis and a cholestatic pattern, including elevated direct bilirubin and alkaline phosphatase levels. The remaining parameters were within normal limits.

Blood analysis		
Investigation	Value	Reference range
Hemoglobin	12.0 g/dl	11.0-18.0 g/dl
Total leukocyte count	20800 cells/cumm	4000-11000 cells/cumm
Serum bilirubin (total)	6.3 mg/dl	0.3-1.2 mg/dl
Serum bilirubin (direct)	5.0 mg/dl	0.0-0.3 mg/dl
Serum bilirubin (indirect)	1.3 mg/dl	0.2-0.8 mg/dl
Alkaline phosphatase	378 U/L	45.00-129.00 U/L

**Figure 1:**
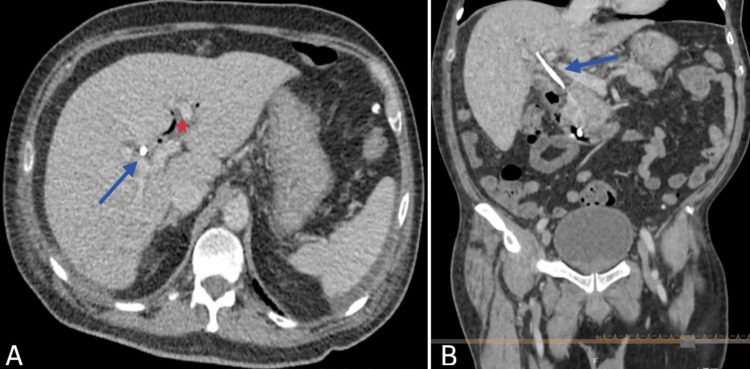
Non-contrast abdominopelvic CT scan. (A) Axial view: The gallbladder is not visualized. The blue arrow indicates hyperdense material within the common bile duct, corresponding to biliary diversion surgical material. The asterisk denotes pneumobilia. (B) Coronal reconstruction: The gallbladder remains absent. The blue arrow highlights a linear hyperdense structure within the bile duct lumen, extending distally toward the second portion of the duodenum. No associated fluid collections are observed.

A contrast-enhanced abdominal and pelvic computed tomography (CT) scan demonstrated the absence of the gallbladder, presence of a biliary stent, and dilation of the common bile duct (Figure 1).

**Figure 2:**
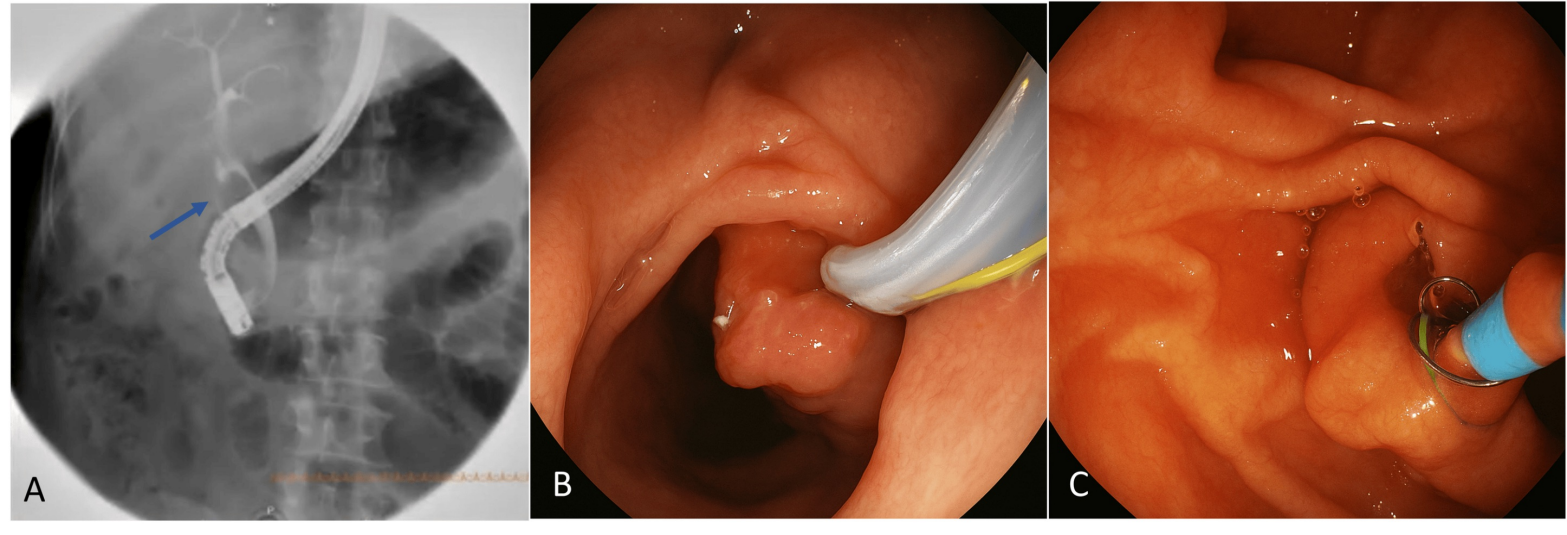
ERCP findings confirming gallbladder agenesis and choledocholithiasis. (A) Cholangiographic image showing the absence of the gallbladder. The blue arrow indicates a 20 × 20 mm filling defect in the supraduodenal portion of the common bile duct, consistent with choledocholithiasis. (B) Endoscopic view of the ERCP probe at the level of the duodenal papilla. (C) Extended sphincterotomy and placement of a 10 Fr × 9 cm plastic biliary stent. ERCP: endoscopic retrograde cholangiopancreatography.

Based on clinical and laboratory findings, a diagnosis of moderate cholangitis was established according to the American Society for Gastrointestinal Endoscopy (ASGE) criteria. Empirical antibiotic therapy with piperacillin/tazobactam was initiated, and an ERCP was performed (Figure 2).

**Figure 3:**
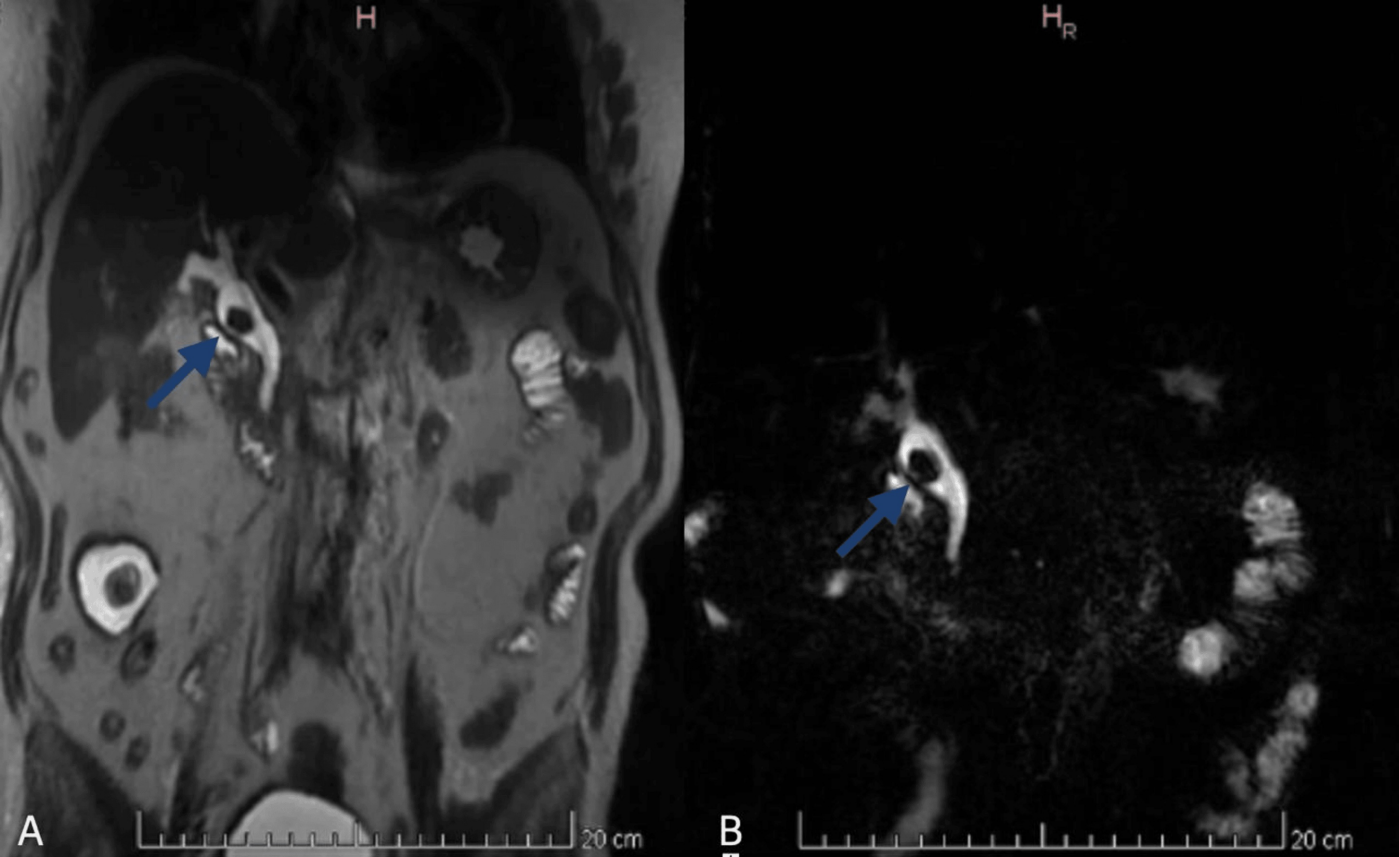
Magnetic resonance cholangiopancreatography (MRCP). Coronal MRCP image showing the absence of the gallbladder and cystic duct. A single 20 mm filling defect is observed within the common bile duct (blue arrow), consistent with a gallstone.

Given inconclusive ERCP results due to a large common bile duct stone, magnetic resonance cholangiopancreatography (MRCP) was requested for better anatomical characterization. MRCP confirmed gallbladder agenesis and choledocholithiasis (Figure 3).

Therapeutic intervention

**Figure 4:**
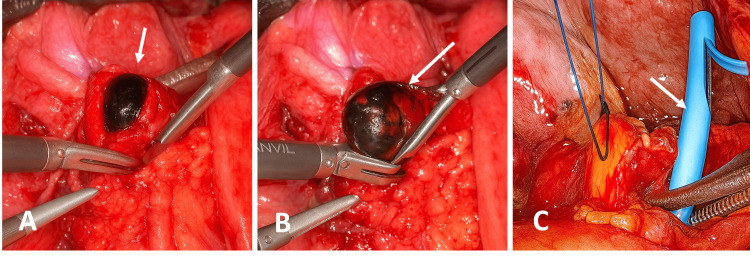
Laparoscopic common bile duct exploration. (A) Intraoperative view showing a gallstone lodged in the common bile duct. (B) Extraction of a single bile duct stone measuring 20 × 15 mm. (C) Retrieved biliary endoprosthesis following stone removal.

Due to the inconclusive ERCP and persistent biliary obstruction, laparoscopic biliary tract exploration was undertaken. Intraoperatively, gallbladder agenesis was confirmed along with a dilated common bile duct. A single biliary stone measuring 20 x 15 mm and the previously placed stent were successfully removed. Intraoperative cholangiography showed unobstructed contrast flow without filling defects (Figure 4).

Monitoring and results

The patient’s postoperative course was uneventful. He was discharged three days after surgery with normalization of bilirubin levels and no signs of bile leakage or other complications.

## Discussion

Gallbladder agenesis (GA) is a congenital anomaly that arises during the fourth week of gestation due to the failure of development or canalization of the cystic bud. The cystic duct normally forms during the seventh week of gestation [1]. This condition is more prevalent in women, with a female-to-male ratio of approximately 3:1, and is most commonly diagnosed during the third decade of life. GA may be associated with other congenital anomalies, particularly involving the gastrointestinal, genitourinary, or musculoskeletal systems [2]. In our case, the patient also had a congenital musculoskeletal malformation of the right upper limb.

Approximately 50% of patients with GA remain asymptomatic throughout life. However, the other half may present with symptoms mimicking cholelithiasis, such as right upper quadrant abdominal pain (reported in up to 90% of cases), nausea and vomiting (66%), intolerance to fatty foods (37%), and dyspepsia (30%) [2-4]. In some cases, GA may even manifest as choledocholithiasis, likely due to biliary dyskinesia or sphincter of Oddi dysfunction resulting from sphincter hypertonia. This mechanism is consistent with the presentation in our patient.

Ultrasound is typically the first-line imaging modality used in evaluating biliary symptoms. However, in cases where the gallbladder is not visualized and the findings are inconclusive, further imaging is essential before proceeding with surgical exploration. According to Malde's diagnostic and treatment algorithm (2010), additional imaging such as CT, ERCP, or MRCP is recommended to clarify the diagnosis and prevent unnecessary surgery [5,6].

Despite advances in imaging, GA remains a diagnostic challenge and is frequently discovered incidentally during surgery, often during attempted laparoscopic cholecystectomy [2,4]. This delay in diagnosis can result in adverse outcomes due to unnecessary surgical dissection. The attempt to identify a non-existent gallbladder and obtain the critical view of safety may increase the risk of iatrogenic bile duct injury due to excessive manipulation [5].

Interestingly, many symptomatic GA patients report relief of symptoms following surgical exploration. It has been hypothesized that this improvement may be due to the release of adhesions in the gallbladder fossa or around the portal structures during the procedure [7].

Given the rarity of GA, no standardized diagnostic or management guidelines currently exist. For patients with mild to moderate symptoms, conservative treatment is often recommended, typically including smooth muscle relaxants (e.g., hyoscyamine) in combination with low-dose tricyclic antidepressants. In more severe or persistent cases, sphincterotomy may be indicated [6,8].

In our case, surgical intervention was not performed to diagnose GA but rather to manage a large common bile duct stone after conventional ERCP failed. Intraoperative findings confirmed the absence of the gallbladder, consistent with GA.

## Conclusions

Gallbladder agenesis is a rare congenital condition that presents a significant diagnostic challenge for surgeons due to its nonspecific clinical manifestations and the limitations of conventional imaging modalities. Although often asymptomatic, it may mimic the presentation of cholelithiasis, potentially leading to unnecessary surgical interventions if not recognized and investigated appropriately. The absence of well-established diagnostic and therapeutic guidelines underscores the need for an individualized approach based on clinical presentation and advanced imaging techniques.

Conservative management is recommended for patients with mild symptoms, while surgical or more invasive procedures should be reserved for specific complications, such as choledocholithiasis. In this case, although surgical intervention was necessary due to associated biliary pathology, it reinforces the importance of accurate preoperative diagnosis to minimize surgical risks and prevent iatrogenic injury.
